# Applying Metabolomics to Understand the Aggressive Phenotype and Identify Novel Therapeutic Targets in Glioblastoma

**DOI:** 10.3390/metabo4030740

**Published:** 2014-08-27

**Authors:** Kamran A. Ahmed, Prakash Chinnaiyan

**Affiliations:** Department of Radiation Oncology, H. Lee Moffitt Cancer Center and Research Institute, Tampa, FL 33612, USA; E-Mail: kamran.ahmed@moffitt.org

**Keywords:** metabolomics, glioblastoma, metabolites

## Abstract

Glioblastoma continues to be an invariably fatal malignancy. The established approach for understanding the biology of these aggressive tumors in an effort to identify novel molecular targets has largely been genotype-based. Unfortunately, clinical gains offered by this level of understanding have been limited, largely based on the complex nature of signaling networks associated with tumorigenesis and the inability to delineate the key “functional” signaling pathways actually driving growth in an individual tumor. Metabolomics is the global quantitative assessment of endogenous metabolites within a biological system, taking into account genetic regulation, altered kinetic activity of enzymes, and changes in metabolic reactions. Thus, compared to genomics and proteomics, metabolomics reflects changes in phenotype and therefore function. In this review, we highlight some of the key advancements that have been made in applying metabolomics to understand the aggressive phenotype of glioblastoma. Collectively, these studies have provided a previously unrecognized window into the underlying biology of these tumors. Current and future efforts are designed to determine how this technology may be applied to improve diagnosis and predict the aggressiveness of glioblastoma, and more importantly, identify novel, therapeutic strategies designed to improve clinical outcomes.

## 1. Introduction

Glioblastoma (GBM) is the most common and aggressive primary central nervous system neoplasm in adults. Despite advances in surgical resection, adjuvant radiation and chemotherapy, median survival remains between 12 and 15 months [1]. Considerable progress has been made in understanding the underlying biology of gliomas through genotype-based approaches [2]. Unfortunately, clinical gains offered by this level of understanding have been limited, largely based on the complex nature of signaling networks associated with tumorigenesis and the inability to delineate the key “functional” signaling pathways actually driving growth in an individual tumor. While cancers have access to a wide variety of genetic and/or epigenetic modifications, there are a limited number of metabolic strategies that they can employ. Further, compared to the genome and the proteome, metabolism reflects changes in phenotype and therefore function [3,4]. Therefore, a fundamental understanding of metabolic differences between cancer and normal cells may provide insight into novel therapeutic targets in this otherwise incurable malignancy.

Metabolomics is the global quantitative assessment of endogenous metabolites within a biological system, taking into account genetic regulation, altered kinetic activity of enzymes, and changes in metabolic reactions [3,4]. This line of investigation has already identified several key differences in the metabolite profiles between normal cells and cancer, including such malignancies as esophageal, prostate, colon, ovarian, and breast cancer [5,6,7,8,9]. This line of investigation has also extended to the study of malignant gliomas, providing novel biologic insight into the aggressive phenotype of this malignancy. In this study, we review the current literature on the study of altered metabolism in glioma using a variety of techniques, including proton nuclear magnetic resonance (NMR), magnetic resonance spectroscopy (MRS), and global metabolomic profiling. In addition, we highlight the work of our own group in identifying novel metabolomic signatures in GBM and how this technology can be applied to provide insight into previously undescribed metabolic pathways with biologic relevance. 

## 2. The Biology of GBM Metabolism

The study of altered metabolism in GBM has been undertaken using several different techniques. Among these include proton NMR, MRS, and global metabolic profiling. These technological advancements have provided unique insight into the underlying biology of GBMs and each technique has unique applications, with both advantages and limitations.

### 2.1. Proton Nuclear Magnetic Resonance (^1^H NMR)

Proton NMR works by applying a magnetic field to the unknown compound. The atomic nucleus generates a magnetic field. When an external magnetic field is present, the nuclei align themselves either with or against the external field. When enough energy is applied to the atom, its spin state will flip. The nucleus then undergoes relaxation when it returns to its original state. The proton NMR spectrometer reads these signals and graphs signal frequency *vs.* intensity, generating a plot that allows for the determination of a chemical compound against a reference compound. NMR identifies the unique make-up of a compound by distinguishing the carbon and hydrogen framework of an organic structure. This technique allows for the determination of the entire molecular structure of a molecule.

NMR analysis has been conducted for a number of years on brain tumor cells. Some of the initial work was performed in the early 1990s by Florian *et al.* [10]. Using ^1^H NMR spectroscopy and high performance liquid chromatography, this group identified metabolic differences in several central nervous system tumors, including meningiomas, neuroblastomas, and GBMs. The study found that spectra from meningiomas featured relatively high signals from alanine. Intense signals from creatine were present in neuroblastoma spectra. However, these signals were not present in GBM. Additionally, the study found statistically significant differences by ^1^H NMR spectroscopy in the amounts of alanine, glutamate, creatine, phosphorylcholine, and threonine among the types of tumors examined. These findings support unique metabolic programs are driving phenotypically different malignancies.

Cuperlovic-Culf *et al.*, reported on the results of metabolite extracts from a selection of nine GBM cell lines using ^1^H NMR analysis [11]. The study found differences in metabolic markers, including choline, lactate, and glutamine between GBM groups. They went on to identify four metabolic subtypes from these lines tested, each corresponding to a unique transcriptional signature determined by gene expression profiles.

In addition, the levels of several amino acids have been shown to differ between grades of oligodendroglioma. Erb *et al.*, reported on the metabolomic profiling of 34 brain biopsies of low-grade (*n* = 10) and high-grade (*n* = 24) oligodendrogliomas using high-resolution magic angle spinning nuclear magnetic resonance spectroscopy [12]. This study found that the most discriminant grade-specific metabolites were alanine, lipids, valine, total choline compounds, proline, myoinositol, taurine, glutamine, glutamate, γ-aminobutyric acid (GABA), malate, N-acetyl-aspartate (NAA), acetate, and creatine. The study further found that there was an increase in amino acid production via nonoxidative pathways in high-grade oligodendrogliomas, demonstrating a shift towards fermentative metabolism associated with high-grade tumors, a finding that has been reported previously in the literature [13,14].

Kauppinen and colleagues have quantified ^1^H NMR visible lipids *in vivo* and characterized their biophysical and biochemical nature in gliomas during programmed cell death (PCD) both *ex vivo* and *in vitro* [15]. The study found that the concentration of polyunsaturated fatty acids increased three fold during PCD. The group identified CH=CH and CH=CHCH2CH=CH as the most significant in monitoring the dynamics of PCD. Furthermore, their work has shown that both *in vivo* and *in vitro* glycine and creatine concentrations followed cell density, whereas choline containing compounds were unaffected by cell loss [16]. Meanwhile, both saturated and unsaturated ^1^H NMR visible lipids increased. However, choline containing compounds were unaffected by a decrease in cell density. Taken together, the work of the Kauppinen group indicates the potential for ^1^H NMR to track the progress of cell death and drug efficacy in glioma cells.

Guidoni *et al.*, recently reported on the results of GBM stem-like cells (GSC) using proton NMR [17]. GSC lines generated from tumors close to the subventricular zone of the temporal lobe were compared with those generated from neural stem/progenitor cells from the adult olfactory bulb. The study found distinct fingerprints from GSCs with signals from myoinositol (Myo-I), UDP-hexosamines (UDP-Hex) and glycine indicating an astrocyte/glioma metabolism. In addition, there appeared to be metabolic heterogeneity between the GSC lines. For example, strong signals from N-acetyl aspartate, GABA, and creatine, suggestive of a neuronal fingerprint were noted in one line, yet absent in another. In addition, a strong lipid signature was also noted in a specific GSC line, consistent with astrocytic/glioma-like metabolism. The authors went on to extend these investigations in a panel of GSC lines, validating these unique metabolic subtypes in GSC.

### 2.2. Magnetic Resonance Spectroscopy (MRS)

MRS is an *in vivo* technique similar to the more commonly utilized method of MRI scanning. MRS allows for the study of metabolic changes in the brain in conditions such as Alzheimer’s disease, seizure disorders, and brain tumors. Unlike MRI, which uses signals from hydrogen atoms in water to create images of the brain, MRS uses these signals to determine the relative concentrations of target brain metabolites. Since MRS is an imaging approach, it is noninvasive, therefore the key advantage of this technique is that it can be used to assess patients through treatment. Although MRS can be used to identify single metabolites *in vivo*, the limited number of metabolites that can be identified by MRS makes global studies of the metabolome difficult. Several groups have identified unique metabolite signatures in gliomas utilizing MRS.

A study by Yamasaki *et al.*, in 2005, found that low and high-grade gliomas showed differences in production of lactate and lipids [18]. The study utilized single-voxel proton MRS in 213 patients, including 163 patients with brain tumors. The study separated these patients into negative, positive, and strongly positive for lactate and lipids. The study found significant differences between lactate and lipid levels between World Health Organization (WHO) grades 2 and 3 and between grades 3 and 4 tumors, underscoring the importance of these metabolic signaling pathways in the underlying aggressive phenotype of this malignancy. Furthermore, the study found that lipid levels were a more significant factor for the discrimination between WHO grades 2 and 3 and between grades 3 and 4. The study concluded that there exists a significant correlation between lactate and lipid expression and the WHO grade of tumors. A similar finding was noted by a German group utilizing MRS [19]. Choline serves as an important compound in the formation of cell membranes and elevated levels signify increased cell turnover and proliferation. The study found that compared to non-neoplastic lesions, tumors demonstrated elevated levels of choline, with decreased levels of creatine and N-acetyl-aspartate (NAA). With higher WHO tumor grading, gliomas were found to have significantly increased choline and lipid formation. A similar finding was seen by Dowling *et al.*, in a report of 29 patients who underwent 3D proton MRS before undergoing surgery [20]. When a pattern of increased choline and decreased NAA was noted, biopsy specimens were invariably positive for tumor. When choline and NAA resonances were low, findings on surgical biopsy were more variable and less likely to be tumor.

A study by Castillo *et al.*, assessed differences in levels of Myo-I between cerebral astrocytomas of various grades [21]. Myo-I is involved in protein C kinase activation and is found in astrocytes [22]. Protein C kinase leads to the production of proteolytic enzymes and a greater abundance of these are found in more aggressive primary tumors. The levels of Myo-I have been shown to be helpful in predicting histologic grade of tumors [23] The study assessed five control subjects, 14 patients with low grade astrocytomas, 10 patients with anaplastic astrocytomas, and 10 patients with GBMs using single volume proton MRS. Levels of Myo-I/creatine were higher (0.82 +/− 0.25) in patients with low-grade astrocytoma, intermediate (0.49 +/− 0.07) in control subjects, and lower in patients with anaplastic astrocytoma (0.33 +/− 0.16) and GBM (0.15 +/− 0.12). The authors concluded that levels of Myo-I might have important implications in the presurgical grading of brain tumors derived by proton MRS.

### 2.3. Mass Spectrometry

Metabolomics is the global quantitative assessment of endogenous metabolites within a biologic system. A clear advantage of this technique is that it allows for a comprehensive understanding of the entire metabolome of a given system, therefore, it is an ideal assay for discovery-based approaches. Although, this method is rich with the output of information, a limitation of this approach is that it requires tissue and, therefore, cannot be utilized as MRS and only provides a static picture into a system’s underlying metabolism. This technique requires sample preparation and access to techniques such as liquid and gas chromatography, mass spectroscopy, a large panel of reference compounds, and considerable bioinformatics support.

As described above, a unique application of global metabolomic profiling is in the context of metabolism-based discovery. An example of this involves the discovery of the novel oncometabolite 2-hydroxyglutarate (2-HG) in IDH1 mutated glioma [24]. This line of investigation went on to further support the elegant interplay between genetic mutation, leading to oncometabolite formation, that in turn demonstrates the capacity to regulate cells at the epigenetic level [25].

Our group took advantage of the high-throughput potential of metabolomics to provide a global perspective on glioma metabolism. Specifically, little is known regarding the relationships between underlying metabolic alterations and mechanisms promoting the observed aggressive phenotype in these tumors. The seminal observation made by Otto Warburg nearly a century ago [26,27], described “aerobic glycolysis”, *i.e.*, a high fermentative metabolism of glucose resulting in production and release of lactic acid, even in the presence of adequate oxygen, yet a definitive explanation for why tumor cells metabolize glucose through the seemingly inefficient process of aerobic glycolysis continues to be elusive. Nonetheless, its relevance to cancer biology is evident with the widespread application of 18-FDG-PET imaging, which can predict histologic grade in glioma with relatively high accuracy. Grade II tumors typically demonstrate low specific uptake values (SUV), while high-grade tumors (Grade III and IV) demonstrate high SUVs [28]. Hence, there are grade-associated changes in glioma metabolism, yet these have not been extensively characterized. Therefore, we performed global metabolomic profiling using liquid/gas chromatography coupled with mass spectrometry on patient derived glioma tissue samples, comparing high and low grade glioma [29]. This line of investigation uncovered numerous metabolic programs differentiating low and high-grade tumors, with significant increase in several metabolites involved in amino acid metabolism, which included glutathione and tryptophan and a decrease in creatine, which is consistent with studies performed using alternate methods described above. One of the most notable involved accumulation of the downstream glycolytic intermediates phosphoenolpyruvate (PEP) and 3-phosphoglycerate, along with increases in metabolites associated with shunting into the pentose phosphate pathway and nucleotide synthesis consistent with anabolic metabolism. Interestingly, random forest analysis identified 2-HG as the top-ranked biochemical for tumor grade classification, further supporting its biologic relevance in these tumors.

These studies provided several additional levels of insight into the underlying biology of GBM. One example involves the identification of a metabolic switch that may underlie a specific subtype of GBM. Specifically, several groups have previously identified malignant glioma subtypes based on transcriptional signatures that provide insight into underlying molecular heterogeneity of these tumors; of these the most aggressive subtype has been termed mesenchymal [30]. Although biologically interesting, this level of understanding has not led to the identification of any subtype specific molecular targets, thereby limiting clinical application. Through our investigation, which was a cross-platform analysis coupling transcriptional signatures with global metabolomic profiles, we identified the accumulation of PEP to highly correlate with the mesenchymal subtype. Therefore, in addition to furthering our understanding of the underlying biology regulating the aggressive nature of this subtype, this supports strategies testing PKM2 activators to increase metabolism of this intermediate as a form of metabolism-based cancer therapy [31].

In addition, we identified 3 unique metabolic subtypes in malignant glioma that were termed energetic, anabolic, and phospholipid catabolism based on the metabolites comprising each subgroup. Through unbiased, hierarchical clustering of the glioma metabolome, metabolites associated with divergence of glycolytic flux, characterized by accumulation of PEP and 3PG, as described above, were termed anabolic. Within the anabolic subtype, which again was consistent with GBM, we identified a unique, particularly aggressive metabolic subgroup with a signature consistent with increased phospholipid catabolism. This mainly consisted of decreased accumulation of several GPCs with no associated increase in phosphocholine. Interestingly, altered phospholipid catabolism has been previously described in glioma, with higher PCHo/GPC ratios found in the ^1^H spectra of human GBM when compared to lower grade tumors [32]. In addition to insight into the underlying biology of glioma, these identified metabolic subtypes appear to also provide information on the aggressiveness of an individual tumor, with the phospholipid subtype portending a worse prognosis. Surprisingly, these identified metabolomic subgroups appeared to be independent from both previously recognized malignant glioma subgroups and transcriptional profiles. This suggests that other global processes as genomic and/or epigenetic regulation, including EGFR amplification or mutation and PTEN activation, may be driving the observed metabolomic subtypes and that integrating these platforms may provide further insight into the signaling processes driving the observed metabolic phenotype.

Lastly, through this line of investigation, we identified a previously undescribed metabolic pathway involved in GBM involving cysteine catabolism. There was more than a 23-fold increase in the metabolic intermediate cysteine sulfinic acid (CSA) in GBM, ranking it as the metabolite with the highest relative accumulation when compared to low-grade glioma [33]. We went on to identify functional consequences of pathway activation, and more importantly, demonstrated that attenuating this pathway using stable shRNA-mediated constructs targeting CSA’s biosynthetic enzyme cysteine dioxygenase 1 (CDO1) decreased GBM growth in preclinical models. Although seminal work involving IDH1 mutations and the onco-metabolite 2-HG have been identified in low-grade glioma, as described above [24], no clear metabolic pathways specific to aggressive high-grade glioma have yet been described. The relevance of cysteine metabolism in cancer has gained considerable interest in recent years. The generation of glutathione from cysteine uptake via the system x_c_^−^ transporter, that exchanges glutamate with cysteine, and its associated role in modulating redox status, is well established in tumors [34]. Further, the glutamate exchanged during this process appears to also provide gliomas with a survival advantage, causing excitotoxic death of neurons in the vicinity of the tumor [35]. Additionally, the system x_c_^−^ transporter itself is currently being investigated as a novel, tumor-specific imaging target, demonstrating the capacity to localize to areas of oxidative stress [36]. Our findings, which identify co-option of cysteine metabolism towards this alternate, parallel pathway resulting in CSA synthesis in aggressive brain tumors provides a previously unrecognized level of insight into the contributory role this emerging metabolic pathway has in gliomagenesis.

## 3. Metabolomics and Cerebrospinal Fluid (CSF)

In addition to metabolomically profiling a tumor, the study of metabolomics has also been assessed in the CSF of patients with gliomas. This line of investigation may extend the application of metabolomics, as CSF and/or plasma may be more readily available than tumor tissue, and therefore, may allow for assessment of therapeutics response, and/or changes in biology underlying tumor recurrence. A study from Kobe University in Japan assessed the CSF of 32 patients with histologically confirmed gliomas using gas chromatography/mass spectrometry [37]. The citric acid levels were significantly higher in GBM samples than in grade I-II tumors (1.77 fold *p* = 0.0125) and grade III (1.83 fold *p* < 0.0174). The same was true for isocitric acid in GBM samples compared to grades I-II (1.78 fold *p* = 0.0096) and grade 3 (1.83 fold *p* < 0.0174). In addition, the lactic and 2-aminopimelic acid levels were relatively higher in the GBM samples than in the grade I-II glioma samples. The study concluded that these CSF metabolite levels may be useful in predicting glioma malignancy and prognosis. A study by Locasale *et al.*, analyzed CSF metabolic profiles from 10 patients with malignant gliomas using liquid chromatography/mass spectrometry [38]. The relative levels of 124 polar metabolites were profiled. The study found significant differences in CSF metabolite composition between malignant gliomas. Newly diagnosed patients were found to exhibit low levels of metabolites involved in tryptophan metabolism. Taken together, these studies provide evidence that metabolic analysis can be conducted on CSF for future research and potential clinical trials.

## 4. Metabolomics and Treatment Response

As described above, several studies to date have identified key differences in the metabolome of gliomas based on tumor grade and subtype. Another application of metabolomics is to evaluate these changes in the metabolome to assess the response to treatment. Wibom *et al.*, reported on the extracellular fluid of patients who received radiotherapy for the treatment of GBM in order to identify early responders to treatment [39]. The study utilized stereotactic microdialysis catheters to sample extracellular fluid from glioblastoma. Microdialysis catheters were implanted in the contrast enhancing tumor as well as brain adjacent to the tumor. The study found metabolic changes in radiotherapy treated samples both in the tumor and brain adjacent to tumor samples with receiver operating characteristic (ROC) values of 0.896 and 0.821, respectively. A less invasive metabolomic test was used by Tandle *et al.*, utilizing urine samples from patients with GBM [40]. Of the 46 samples showing significant differences between the samples from GBM and healthy controls, the levels of 6.5% showed a difference in patient survival. These included mannitol, pyroglutamine, and 7-methylguanine. In addition, several compounds were shown to have significant differences in levels, based on whether patients received RT or not. These included N-acetylated metabolites including N-acetylphenylalanine, N-acetyltryptophan, N-acetyltyrosine, and N-acetylproline, which were all significantly elevated in the urine of patients that received RT. In addition, TCA cycle intermediates including citrate, iso-citrate, alpha-ketoglutarate, succinate, fumarate, malate, and 2-hydroxyglutarate were also significantly elevated post-RT. The elevation of metabolites involved in the TCA cycle may be secondary to oxidative stress caused by radiation. This is consistent with the data of Wibom *et al.*

## 5. Conclusions

GBM is the most common and most aggressive brain tumor. Despite years of research and clinical trials, median survival remains poor [1]. Technological advancements in studying metabolism through metabolomics have provided an unprecedented insight into the underlying biology that may be driving the aggressive phenotype of these tumors. Current and future efforts are designed to determine how this technology may be applied to improve diagnosis and predict the aggressiveness of glioblastoma, and more importantly, identify novel, therapeutic strategies designed to improve clinical outcomes.
